# Genome-wide functional annotation of variants: a systematic review of state-of-the-art tools, techniques and resources

**DOI:** 10.3389/fphar.2025.1474026

**Published:** 2025-03-03

**Authors:** Eleftherios Pilalis, Dimitrios Zisis, Christina Andrinopoulou, Theodora Karamanidou, Maria Antonara, Thanos G. Stavropoulos, Aristotelis Chatziioannou

**Affiliations:** ^1^ e-NIOS Applications PC, Kallithea, Greece; ^2^ Pfizer Center for Digital Innovation, Thessaloniki, Greece; ^3^ Biomedical Research Foundation of the Academy of Athens, Athens, Greece

**Keywords:** genome-wide, variant annotation, genomic variation, whole-genome sequencing, intergenic, GWAS, genotyping, whole-exome sequencing

## Abstract

The recent advancement of sequencing technologies marks a significant shift in the character and complexity of the digital genomic data universe, encompassing diverse types of molecular data, screened through manifold technological platforms. As a result, a plethora of fully assembled genomes are generated that span vertically the evolutionary scale. Notwithstanding the tsunami of thriving innovations that accomplish unprecedented, nucleotide-level, structural and functional annotation, an exhaustive, systemic, massive genome-wide functional annotation remains elusive, particularly when the criterion is automation and efficiency in data-agnostic interpretation. The latter is of paramount importance for the elaboration of strategies for sophisticated, data-driven genome-wide annotation, which aim to impart a sustainable and comprehensive systemic approach to addressing whole genome variation. Therefore, it is essential to develop methods and tools that promote systematic functional genomic annotation, with emphasis on mechanistic information exceeding the limits of coding regions, and exploiting the chunks of pertinent information residing in non-coding regions, including promoter and enhancer sequences, non-coding RNAs, DNA methylation sites, transcription factor binding sites, transposable elements and more. This review provides an overview of the current state-of-the-art in genome-wide functional annotation of genetic variation, including existing bioinformatic tools, resources, databases and platforms currently available or reported in the literature. Particular emphasis is placed on the functional annotation of variants that lie outside protein-coding genomic regions (intronic or intergenic), their potential co-localization with regulatory element areas, such as putative non-coding RNA regions, and the assessment of their functional impact on the investigated phenotype. In addition, state-of-the-art tools that leverage data obtained from WGS and GWAS-based analyses are discussed, along with future bioinformatics directions and developments. These future directions emphasize efficient, comprehensive, and largely automated functional annotation of both coding and non-coding genomic variants, as well as their optimal evaluation.

## 1 Introduction

Despite the rapidly increasing number of Whole Genome Sequencing (WGS), Whole Exome Sequencing (WES) (Lappalainen et al., 2019), and Genome-Wide Association Studies (GWAS) (Uffelmann et al., 2021; Visscher et al., 2017), as well as significant advancements in processing of diverse types of molecular data provided by powerful sequencing technologies, their exhaustive and massive genome-wide annotation remains far from optimal and automated (Salzberg, 2019; Zerbino et al., 2020). Still, the efforts committed to systematic genomic annotation thus far have been substantial, providing an unprecedented volume of mechanistic information about a wide range of functional elements, including promoter and enhancer sequences, non-coding RNAs, DNA methylation sites, transcription factor binding sites (TFBS), transposable elements and other.

Functional annotation of genetic variants is a critical step in genomics research, enabling the translation of sequencing data into meaningful biological insights. The major types of genetic variation include Single Nucleotide Variants (SNVs) and small insertions or deletions (indels) of two or more nucleotides, often detected through WES or WGS at the individual level. Single Nucleotide Polymorphisms (SNPs) also refer to changes in a single nucleotide in a DNA sequence, although they represent frequent variations in the genome shared across a population, typically identified through GWAS. While the concept of variant identification involves the detection of the precise location of variants on the reference genome and determining the alternate alleles, functional annotation specifically refers to predicting the potential impact of these variants on protein structure, gene expression, cellular functions, and biological processes. This process involves several key stages, each leveraging advanced computational tools and integrative approaches to elucidate the roles of genetic variants in health and disease.

Nonetheless, the ability of WGS/WES and GWAS in causally associating genetic variation with disease is hindered by a number of significant limitations and challenges. For instance, Linkage Disequilibrium (LD), referring to the non-random association of alleles at two or more loci in a population, causes certain combinations of SNP genotypes to occur together more or less frequently than would be expected by chance. As a result, true causal variants may be found among numerous confounding variants that are irrelevant to the disease but they are just colocalized in the genome. This limitation is even more crucial for polygenic disorders, caused by the combined effect of multiple variants, as each single causal variant is expected to have a small contribution. High-resolution, fine-mapping techniques help to narrow down the set of candidate variants and determine which variants in a genomic region are most likely to be causally related to a complex trait after accounting for how the variants in the region are correlated reviewed in (Schaid et al., 2018).

Another major challenge lies in the fact that the majority of human genetic variation resides in non-protein coding regions of the genome. The challenge of exploring non-coding regions (intergenic, intronic) and providing exhaustive functional annotation of these unknown regions remains substantial, despite the critical role that non-coding regions play in human disease (Zhang and Lupski, 2015). Nonetheless, the crux of a mechanistically insightful genome annotation lies in the functional interpretation at the gene level, rendering the interpretation of intergenic and non-coding variants particularly difficult. The expanding collection of human WGS data, combined with the understanding of regulatory elements such as promoters, enhancers, TFBS, non-coding RNAs, and transposable elements, has the potential to transform our limited knowledge of the functional importance of these regions into a wealth of information. In addition, genomic regulation studies benefit from advanced sequencing techniques such as Hi-C (Lieberman-Aiden et al., 2009), which give insights to the three-dimensional (3D) organization of the genome and maps global physical interactions between different genomic regions. Thus. Hi-C is able to map long-range interactions by identifying physical contacts between regulatory elements and gene promoters in 3D space. These advancements will enable researchers to explore non-coding regions of the human genome, unearthing valuable insights with significant implications for various diseases and pathologies.

By providing methods and resources for comprehensive functional annotation of both coding and non-coding regions, we can enhance our understanding of the relationship between non-coding variation and clinical disease. This, in turn, will provide a more thorough understanding of disease biology. Additionally, it could reveal opportunities for developing novel therapeutic targets, generating novel druggable biomarkers, and identifying new drug candidates.

This review discusses the state-of-the-art tools that can leverage WGS and GWAS-based analyses (Mortezaei and Tavallaei, 2021). It provides insights into the specific regulatory elements involved in the functional annotations of non-coding regions and offers guidance on existing data resources that can be utilized to achieve comprehensive, largely automated functional annotation of intergenic or intronic genomic variants.

## 2 Methodology

### 2.1 Search strategy

A systematic literature search was conducted to identify articles focusing on primary publications on tools and resources for genome-wide functional annotation of variants. The search was performed in PubMed, up to June 2024, including only papers in English language. Keywords used in the search included “genome-wide annotation,” “functional annotation,” “genetic variants,” “GWAS, “Whole Genome Sequencing,” and “bioinformatic tools.” The search strategy was designed to include both broad and specific terms to ensure comprehensive coverage.

### 2.2 Inclusion and exclusion criteria

Articles were included if they met the following criteria:

1. Focused on functional annotation of genetic variants.
2. Introducing novel bioinformatics tools or methods for annotation, or comprehensive resources with functional information.
3. Published in the last 10 years or cited more than 50 times.

Exclusion criteria:

1. Articles not providing original data (e.g., reviews, editorials).
2. Articles focused on statistical analysis of variants, rather than functional annotation.
3. Articles published more than 10 years ago or cited less than 50 times.

The restriction on the time frame and number of citations is justified by this review’s scope, which is not to be exhaustive but to highlight the most recent and most commonly used state-of-the-art tools and resources.

**FIGURE 1:**
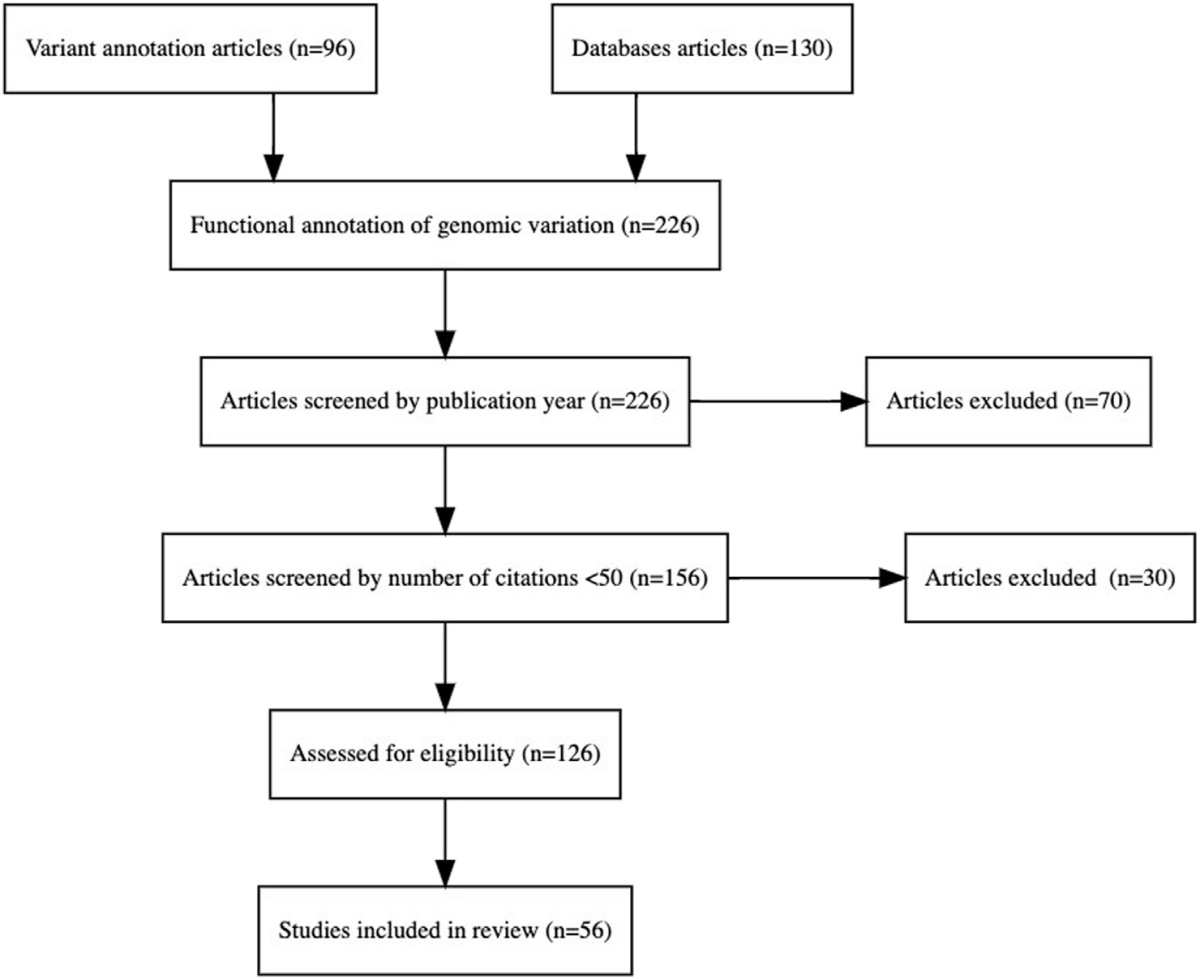
PRISMA flowchart that shows the step-by-step process of the application of inclusion and exclusion criteria to generate a final number of articles for analysis in the literature review.

The PRISMA (Page et al., 2021) flowchart in Figure 1 describes the systematic methodology followed in the selection of studies/articles for the literature review. The PRISMA flowchart ensures transparency and reproducibility in the selection process.

The selection process, as depicted in the flowchart, includes the following steps:

1. Identification: Relevant articles related to the functional annotation of genomic variants were identified from two primary sources: 96 articles focused on variant annotation and 130 articles on databases, amounting to a total of 226 articles for further screening.
2. Screening: The initial screening was conducted based on publication year, resulting in the exclusion of 70 articles that did not meet the temporal criteria. In the second phase of screening the remaining 156 articles were assessed based on their citation counts, with a threshold set at fewer than 50 citations. This phase excluded 30 articles, thereby ensuring the inclusion of more influential and widely recognized studies.
3. Eligibility: The final 126 articles underwent a rigorous evaluation to ensure they met the predefined inclusion criteria for the review. After a comprehensive review and assessment, 56 studies were included in the final review. These studies provide significant insights into the functional annotation of genomic variation and meet all the eligibility criteria established for the review process.

**FIGURE 2:**
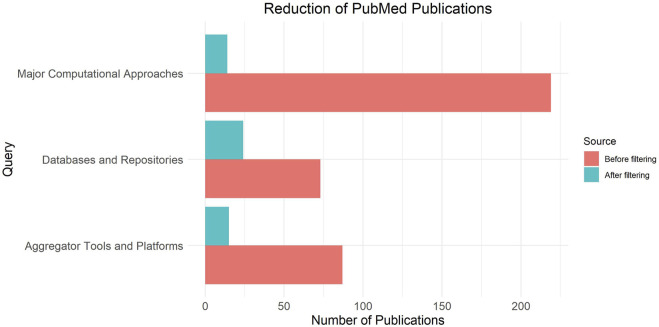
Selection of publications attributed to three distinct categories, namely major computational approaches (fundamental annotation tools), databases and repositories (annotation resources) and aggregator tools and platforms.

Figure 2 presents the number of publications initially found and after selection, attributed to three distinct categories, namely major computational approaches, aggregator tools and platforms, and databases and repositories.

## 3 Computational resources for functional annotation of genetic variants

Variant calling is the process of identifying genetic variants from sequencing data, resulting in an unannotated file, typically in Variant Calling Format (VCF), that contains raw variant positions and allele changes. The initial annotation step involves processing this file with tools that map these variants to genomic features such as genes, promoters, and intergenic regions. This procedure is commonly performed using tools like Ensembl Variant Effect Predictor (VEP) (McLaren et al., 2016) and ANNOVAR (Wang et al., 2010), which can directly handle raw VCF files and are well-suited for large-scale annotation tasks, such as whole-genome and whole-exome sequencing projects.

The landscape of variant annotation tools is quite complex, as different tools target different genomic regions and perform different types of analyses. Some tools specialize in annotating exonic (protein-coding) regions, focusing on variants that may alter amino acid sequences and affect protein function or structure. These tools provide insights into the potential pathogenicity of missense mutations and other coding variants. Other tools concentrate on non-exonic intragenic regions, such as introns and untranslated regions (UTRs), as well as intergenic regions located between genes. These tools often emphasize the identification of regulatory elements, TFBS, and other features that can influence gene expression and regulation. Additionally, there are comprehensive tools that annotate variants across all genomic regions, providing a wide array of annotations including functional impact predictions, conservation scores, regulatory annotations, and disease associations.

A less represented category includes tools that are designed to assess the cumulative impact of multiple variants, analyzing their collective effect on genes, pathways, or biological processes, which is particularly important for understanding complex traits and polygenic diseases. These tools may perform gene-set or pathway analyses, integrating genetic data with functional genomics or GWAS data, to elucidate the broader biological significance of variant combinations.

Additionally, a key distinction exists between tools that use fundamental methodologies to predict the impact of individual variants and aggregator tools, which rely on these foundational tools to perform large-scale variant annotations. For this purpose, Table 1 provides a summary of major computational approaches for predicting the impact of variants, while Table 2 summarizes the main aggregator tools and platforms and their genomic regions of application.

**TABLE 1:** Summary of major computational approaches for functional variant annotation.

Tool	Genomic regions	Description	Relevant aggregator tool	Citations
PolyPhen-2 (Adzhubei et al., 2010)	Exonic	Predicts impact of amino acid substitutions on structural features of protein (Protein-coding)	ANNOVAR, VEP, SnpEff, ShAn	>6000
SIFT (Kumar et al., 2009)	Exonic	Predicts deleterious AASs based on sequence homology and the physical properties of amino acids (Protein-coding)	ANNOVAR, VEP, SnpEff, ShAn	>6000
GERP++ (Davydov et al., 2010)	Intergenic; Exonic; Non-Exonic Intragenic	Uses maximum likelihood evolutionary rate estimation, to identify nucleotide- and element-level scores from multiple sequence alignments	VEP, ANNOVAR	>2000
MutationTaster2/MutationTaster 2021 (Steinhaus et al., 2021)	Exonic; Non-Exonic Intragenic	Machine-Learning classifier to predict disease-causing scores of genomic variants	VEP, ANNOVAR, SnpEff	>2000
CADD (Combined Annotation Dependent Depletion) (Kircher et al., 2014)	Intergenic; Exonic; Non-Exonic Intragenic	Integrates multiple annotations to provide a single score (C-score) indicating the deleteriousness of variants. (Protein-coding)	ANNOVAR, VEP, OpenCRAVAT, ShAn, FUMA	>1,500
RegulomeDB (Boyle et al., 2012)	Intergenic; Non-Exonic Intragenic	Scores variants in non-coding regions based on the existence/overlap of regulatory elements (TFBS, DNase hypersensitivity sites, and histone modifications)	ANNOVAR, VEP, FUMA	>700
REVEL (Ioannidis et al., 2016)	Exonic	Integrates ensemble methods to predict the pathogenicity of rare missense variants by combining multiple scores (PolyPhen-2, SIFT, etc.). (Protein-coding)	VEP, ANNOVAR	>500
FATHMM (Shihab et al., 2013)	Exonic; Non-Exonic Intragenic; Intergenic	Predicts the functional consequences of both coding and non-coding variants in the human genome based on Hidden Markov Models. - Predicts deleterious AASs	ANNOVAR, VEP, SNPnexus	>500
MetaSVM (Dong et al., 2015)	Exonic	A scoring system that Combines the output of multiple individual methods (PolyPhen-2, SIFT, etc.) to predict pathogenicity- High performance in benchmarks. (Protein-coding)	ANNOVAR	>400
DANN (Quang et al., 2015)	Intergenic; Exonic; Non-Exonic Intragenic	Similar to CADD, but with a different ‘nonlinear’ machine learning approach (deep neural networks). Provides D-Scores to predict deleteriousness	VEP, ANNOVAR	>300
Eigen (Ionita-Laza et al., 2016)	Intergenic; Exonic; Non-Exonic Intragenic	Integrates functional genomic annotations into an eigenvector to predict deleteriousness based on a spectral approach (unsupervised learning). (Protein-coding)	Custom Pipelines and Workflows	>200
GenoCanyon (Lu et al., 2015)	Intergenic; Exonic; Non-Exonic Intragenic	Scores and evaluates the functional significance of genomic variants based on Bayesian hierarchical model	Custom Pipelines and Workflows	>200
LINSIGHT (Huang et al., 2017)	Intergenic; Non-Exonic Intragenic	A linear model for estimating negative selection, scoring and identifying non-coding variants indicating functional importance	Custom Pipelines and Workflows	>100

**TABLE 2:** Summary of aggregator tools and platforms.

Tool	Description	Application	Resolution of functional annotation	Functional annotation focus	Citations
ANNOVAR (Wang et al., 2010)	Functional annotation of variants, identification of regulatory elements	Command-line, Limited online plugin	Gene level, Region level, Regulatory elements	Exonic, UTR, Intronic, Intergenic	>3000
VEP (McLaren et al., 2016)	Comprehensive genomic variant annotation	Web-based, command-line, REST API	Gene level, Transcript level, Protein function	Exonic, UTR, Intronic	>2000
SnpEff & SnpSift (Cingolani et al., 2012b; Cingolani et al., 2012a)	Annotation of variants on genes and proteins, filtering annotation results	command line	Gene level, Transcript level, Regulatory elements	Exonic, limited in Intronic, UTR, Intergenic	>1,000
MAGMA (de Leeuw et al., 2015)	Gene-based and gene-set analysis of variants	Open-source command line tool	Gene level, Pathway level	Exonic, Intronic, UTR	>800
HaploReg (Ward and Kellis, 2012)	Annotation for variations in non-coding regions. Explores chromatin states, conservation, and regulatory motif alterations within sets of variants	User interface	Regulatory element level, Chromatin state, Protein binding	Intronic, UTR	>500
OpenTargets Genetics (Ghoussaini et al., 2021)	Integrates genetic, genomic, and chemical publicly available data, identifies and prioritizes therapeutic targets for diseases	User interface, REST API	Gene level, Pathway level, Regulatory elements	Exonic, UTR, Intronic, Intergenic	>300
GWAVA (Ritchie et al., 2014)	Predicts the functional impact of non-coding genetic variants and prioritizes them based on their potential functional impact	Web-based	Regulatory elements, Chromatin state, Protein binding	Exonic, Intronic, UTR	>300
VarSome (Kopanos et al., 2019)	A community driven variant annotation platform with extensive database cross-references that empowers variant functional knowledge sharing	Web-based, user interface	Gene level, Pathway level, Regulatory elements	Exonic, Intronic	>300
InterVar (Li and Wang, 2017)	Interprets the clinical significance of genetic variants based on ACMG-AMP guidelines	Command-line, user interface	Gene level, Clinical significance, Protein function	Exonic	>200
FUMA (Watanabe et al., 2017)	Annotation, prioritization and visualization of GWAS variants integrating multiple biological data sources	Web-based, user interface	Gene level, Pathway level, Regulatory elements, eQTL analysis	Exonic, UTR, Intronic, Intergenic (based on annotations obtained from ANNOVAR)	>200
VAT (Habegger et al., 2012)	Functional annotation of variants, integrates data from multiple sources	Command-line, cloud computing environment	Gene level, Region level, Regulatory elements	Exonic, limited in Intronic	>100
VAAST 2.0 (Hu et al., 2013)	Annotation of WGS variants, identification and prioritization of disease-causing variants	Command line	Gene level, Pathway level, Regulatory elements	Exonic, Intronic (limited)	>100
VPMBench (Ruscheinski et al., 2021)	Benchmarking tool for variant pathogenicity predictors	Web-based, Command-line	Pathway level, Variant level	Protein-coding, intergenic	>100
OpenCRAVAT (Pagel et al., 2020)	Variant annotation, prioritization, and analysis integrating multiple data sources and tools	Web-based, Command-line	Gene level, Pathway level, Regulatory elements	Exonic, UTR, Intronic, limited in intergenic	>80
FAVORannotator (Zhou et al., 2023)	Online portal integrating functional information from multiple sources, prioritizing causal variants in coding and non-coding regions	Web-based	Variant level, Regulatory elements, non-coding variants	Protein-coding, intergenic	>50

### 3.1 Gene-level annotation

Polymorphism Phenotyping v2 (Polyphen-2) (Adzhubei et al., 2010) and Sorting Intolerant From Tolerant (SIFT) (Kumar et al., 2009) predict the impact of amino acid substitutions on structural features of the protein and assess whether protein-coding variants are likely to be damaging or deleterious. Meta-Support Vector Machine (MetaSVM) (Dong et al., 2015) is a computational tool designed to predict the deleteriousness of genetic variants, particularly those in coding regions. It employs Support Vector Machine models to prioritize potentially pathogenic variants, by integrating various functional annotation datasets, including sequence conservation scores, protein domain information, and physicochemical properties of amino acids. Rare Exome Variant Ensemble Learner (REVEL) (Ioannidis et al., 2016) is also focused on coding regions and integrates individual variant effect prediction tools such as SIFT and Polyphen-2, providing a consensus prediction.

MutationTaster2 and MutationTaster 2021 (Schwarz et al., 2014) are tools designed to predict the disease-causing potential of genetic variants. They analyze both coding and intragenic non-coding changes by integrating various data sources, including evolutionary conservation, splice-site alterations, protein features, and regulatory elements, to assess the impact of variants on gene function and pathogenicity. The updated MutationTaster2021 version incorporates various improvements to enhance user-friendliness and processing speed, as well as more recent genomic data for enhanced prediction accuracy.

The above-mentioned tools offer a range of functionalities for predicting the impact of genetic variants, each with distinct strengths and weaknesses. They are primarily focused on coding and intragenic regions, specifically predicting the effects of missense variants (amino acid substitutions) on protein function and structure.

### 3.2 Genome-wide annotation

VEP (McLaren et al., 2016), provides predictions of the impact of variants on gene function, including coding changes (e.g., missense, nonsense, synonymous), splicing effects, and regulatory region impacts, such as promoters, enhancers, and untranslated regions (UTRs). It also provides information on the known clinical significance of variants, by integrating data from clinical databases such as ClinVar (Landrum et al., 2014) and COSMIC (Tate et al., 2019). VEP also incorporates data from functional genomics resources such as the ENCODE project, Roadmap Epigenomics and GTEx, as well as population data and allele frequencies from large-scale population studies such as the 1000 Genomes Project (Genomes et al., 2015), ExAC browser (Karczewski et al., 2017) and gnomAD (Karczewski et al., 2020). VEP is optimized for high-throughput analysis and can rapidly process large volumes of data using parallel processing and distributed computing techniques.

ANNOVAR (Wang et al., 2010) is a widely used tool for the large-scale functional annotation of variants. It integrates data from multiple databases such as RefSeq (Pruitt et al., 2014), ENCODE, 1000 Genomes Project, dbSNP (Sherry et al., 2001), ClinVar (Landrum et al., 2014), and provides functional annotations related to gene function, regulatory regions, evolutionary conservation, pathogenicity, and allele frequencies. ANNOVAR also provides variant prioritization in terms of clinical significance and association with diseases and pathogenic phenotypes. Like VEP, ANNOVAR is also focused on large-scale annotation but without attempting systems-level characterization of assessment of the cumulative impact of variants in the context of pathways. As previously discussed, VEP and ANNOVAR are the most commonly used tools for large-scale annotation of VCF files in WGS/WES pipelines. However, it is worth mentioning that the choice between these tools, as well as the choice in regard to the transcripts reference set (Ensembl/Refseq) may significantly impact the results, as substantial differences between annotations have been reported, highlighting the importance of cross-referencing results for critical applications (McCarthy et al., 2014).GERP++ (Davydov et al., 2010) is a powerful tool for identifying evolutionarily conserved elements in the genome, providing valuable insights into the functional significance of genomic regions. Its conservation scores are widely used in the functional annotation of genetic variants, helping to prioritize variants for further study based on their potential impact on biological functions. GERP++ is particularly useful for identifying conserved non-coding elements, which may include regulatory regions such as enhancers or silencers. While GERP++ provides nucleotide-level constraint metrics, gene-level constraint metrics are typically derived from population genomic data. For instance, the Genome Aggregation Database (gnomAD) (Karczewski et al., 2020) offers gene-level constraint metrics by comparing the observed number of loss-of-function (LoF) variants in a gene to the expected number, based on a mutational model that accounts for sequence context, coverage, and methylation.

Other tools capable of annotating non-coding variants, include Combined Annotation Dependent Depletion (CADD) and RegulomeDB. CADD (Kircher et al., 2014) is a widely used computational tool designed to assess the deleteriousness of genetic variants by integrating diverse annotations into a single score. CADD is particularly valuable for prioritizing variants in both coding and non-coding regions of the genome. Deleterious Annotation of genetic variants using Neural Networks (DANN) (Quang et al., 2015) is based on the same training data as CADD but it employs a different, non-linear Machine Learning approach, namely a deep neural network (DNN) to provide robust predictions on the deleteriousness of both coding and non-coding variants. LINSIGHT (Huang et al., 2017) is a computational tool that predicts the functional importance of noncoding genomic regions by integrating evolutionary conservation with functional genomic data, providing nucleotide-level scores that indicate the likelihood of functional significance.

RegulomeDB (Boyle et al., 2012) is a comprehensive resource designed to annotate and interpret regulatory variants in the human genome. It integrates various types of functional genomics data to provide insights into the potential regulatory roles of genetic variants, particularly those located in non-coding regions. It comprises a variant scoring system, where each variant is assigned a score, based on the evidence supporting its regulatory role. The functional data sources include ENCODE (ENCODE Project Consortium, 2012), Roadmap Epigenomics (Bernstein et al., 2010), and GTEx (GTEx Consortium, 2013), which provide information on regulation such as promoters and enhancers, TFBS, chromatin states, and expression quantitative trait loci (eQTL). Eigen (Ionita-Laza et al., 2016) uses a similar approach based on statistical learning.

Functional Analysis through Hidden Markov Models (FATHMM) (Shihab et al., 2013) integrates various functional annotations, including protein domains, sequence conservation data, and known pathogenic variants from databases such as ClinVar (Landrum et al., 2014) and HGMD (Stenson et al., 2003). It uses Hidden Markov Models to provide a probability score for both coding and non-coding variants, indicating the likelihood that a variant is pathogenic.

GenoCanyon (Lu et al., 2015) is a computational tool designed to predict the functional significance of non-coding genetic variants, with a particular focus on intergenic regions. It combines information from various genomic annotations and employs a probabilistic framework to assess the potential regulatory impact of genetic variants. It aims to integrate diverse functional annotations into a single, continuous score. SnpEff (Cingolani et al., 2012b) and SnpSift (Cingolani et al., 2012a) are companion tools for variant annotation, filtering, and interpretation. SnpEff annotates variants based on their impact on genes, including coding changes (missense, nonsense, synonymous), splicing effects, and regulatory region impacts, whereas SnpSift provides additional functionalities for filtering, manipulating and annotating VCF files.

HaploReg (Ward and Kellis, 2012) is a tool for exploring annotations of the non-coding genome, particularly focusing on variants within haplotype blocks, such as candidate regulatory SNPs at disease-associated loci. It utilizes linkage disequilibrium (LD) (Slatkin, 2008) information from the 1000 Genomes Project to visualize linked single nucleotide polymorphisms (SNPs) and small indels. The tool integrates chromatin state and protein binding data from the Roadmap Epigenomics and ENCODE projects, sequence conservation across mammals, and the effects of SNPs on regulatory motifs and gene expression from eQTL studies.

Open Targets Genetics (Ghoussaini et al., 2021) is a comprehensive resource that integrates human GWAS and functional genomics data, including gene expression, protein abundance, chromatin interaction, and conformation data from various cell types and tissues, to establish robust connections between GWAS-associated loci, variants, and likely causal genes (Ghoussaini et al., 2021). Thus, Open Targets Genetics incorporates GWAS, eQTL, pQTL and epigenetics data resources to enable robust statistical associations and prioritization of genes underlying disease causation.

GWAVA (Ritchie et al., 2014) utilizes machine learning algorithms to classify non-coding genetic variants and prioritizes them based on their potential functional impact, incorporating data from resources such as ENCODE and GENCODE (Harrow et al., 2012).

Other useful tools aiming at the functional and clinical interpretation of variants, include InterVar (Li and Wang, 2017), VAT (Habegger et al., 2012), VAAST 2.0 (Hu et al., 2013), OpenCRAVAT (Pagel et al., 2020), FAVORannotator (Zhou et al., 2023), ShAn (Rathinakannan et al., 2020), and platforms that can be used for aggregation of annotations such as VarSome (Kopanos et al., 2019). VPMBench (Ruscheinski et al., 2021) is a benchmarking tool for variant prioritization methods.

Overall, the aforementioned genome-wide annotation tools have overlapping functionalities but diversities in algorithms, reference databases, prediction models and area of focus, making particularly challenging a direct comparison. ANNOVAR and VEP are comprehensive annotation tools designed for high-throughput variant annotation. They integrate a wide array of resources to provide gene-based, region-based, and filter-based annotations, making them ideal for large-scale genomic studies. GERP++, CADD, DANN, Eigen, GenoCanyon, and LINSIGHT generate scores estimating the deleteriousness or functional significance of variants by integrating evolutionary conservation and functional genomic data. Thus, they are particularly useful for prioritizing variants in whole-genome sequencing data but may lack detailed functional annotations. RegulomeDB, HaploReg, and FAVORannotator specialize in annotating non-coding and intergenic variants with regulatory information, which is valuable for studies exploring regulatory elements but may not extensively cover coding regions. SnpEff & SnpSift offer flexible variant effect prediction and filtering capabilities, suitable for incorporating custom annotations, though they may require bioinformatics expertise to use effectively. OpenTargets Genetics causally links genetic variants to traits and diseases, aiding in identifying potential therapeutic targets, but its reliance on existing GWAS and eQTL datasets may limit its applicability to certain studies. GWAVA and FATHMM use machine learning models to predict the functional impact of non-coding variants, helpful for non-coding variant analysis but potentially less interpretable due to the complexity of the models. OpenCRAVAT is a modular, extensible platform that allows for customized annotation workflows, enhancing flexibility but interpretation is subject to user customization. VarSome and InterVar provide clinical interpretation of variants following ACMG guidelines, aiding in clinical decision-making but necessitating cautious interpretation to avoid over-reliance on automated classifications.

### 3.3 Functional annotation combined with pathway analysis

Another category of functional variant annotation tools focuses on evaluating the combined effects of multiple genetic variants, analyzing how they collectively influence genes, pathways, or biological processes. These tools assess the cumulative impact of variant combinations rather than considering each variant in isolation.

Multi-marker Analysis of GenoMic Annotation (MAGMA) (de Leeuw et al., 2015) is designed for the analysis of genome-wide association study (GWAS) data, featuring gene and pathway-based analysis. MAGMA aggregates the effects of SNPs within a gene to assess the overall contribution of that gene to the trait of interest. It computes gene-level *p-*values by considering the association signals of all SNPs within each gene, considering linkage disequilibrium (LD) between SNPs. Then, it evaluates the enrichment of significant genes within predefined biological pathways, such as MSigDB and KEGG. MAGMA functionality is basically gene-based but it can partially incorporate intergenic annotations through extended gene boundaries, custom region definitions, and the integration of functional genomic data that comprise regulatory region-to-gene mappings.

Functional Mapping and Annotation of GWAS (FUMA) (Watanabe et al., 2017) is an integrative web-based platform using information from multiple biological resources to facilitate functional annotation of GWAS results, gene prioritization and interactive visualization. It accommodates positional, expression quantitative trait loci (eQTL) and chromatin interaction mappings, and provides gene-based, pathway and tissue enrichment results. It enables pathway analysis by linking the variants identified in GWAS to biological pathways, thereby providing insights into the underlying biological mechanisms of complex traits and diseases. Gene set and pathway enrichment analyses is performed on prioritized genes identified through its core SNP2GENE process. This process includes mapping SNPs to genes based on positional, eQTL, and chromatin interaction data. FUMA implements MAGMA gene-based analysis and gene-set analysis on the full GWAS input data. Genes prioritized by SNP2GENE or by the user are also tested for overrepresentation in various gene sets in GENE2FUNC process. FUMA incorporates variants located at intergenic regions, by integrating annotations from functional genomic resources. MAGMA and FUMA leverage genome-wide studies which typically evaluate associations of common variants. However, low-frequency and rare variants are known to play an important role in human disease. Thus, a comprehensive tool would ideally combine additional statistical methodologies and strategies in order to account for also rare variants (reviewed in Lee et al., 2012).

## 4 Comprehensive resources of genomic variation

Several key resources and databases facilitate the aggregation, exploration, annotation, and interpretation of variants, providing essential tools for research and clinical applications. These resources are often used as the main reference for variant annotation tools, such as the aforementioned in the previous section. Table 3 summarizes the main databases and resources for large-scale functional annotation of variants.

**TABLE 3:** Summary of existing databases and resources employed for the functional annotation of variants.

Database/Resource	Description	Type	Genome wide tools	Citations
ENCODE (ENCODE Project Consortium, 2012)	The Encyclopedia of DNA Elements, a project to identify all functional elements in the human genome	Functional genomics database	VEP, ANNOVAR, SnpEff & SnpSift, FUMA, OpenTargets Genetics, GWAVA	16000+
1000 Genomes Project (Genomes et al., 2015)	A project to develop and provide a comprehensive resource on human genetic variation	Genetic variation database	VEP, ANNOVAR, SnpEff & SnpSift, InterVar, VarSome	6000+
RefSeq (Pruitt et al., 2014)	A comprehensive, integrated, non-redundant set of sequences, including genomic DNA, transcripts, and proteins	Reference sequences database	VEP, ANNOVAR, SnpEff & SnpSift, InterVar, VAT, VarSome	5000+
GENCODE (Harrow et al., 2012)	A comprehensive database to provide high-quality annotation of gene features on the human and mouse genomes	Gene annotation database	VEP, ANNOVAR, SnpEff & SnpSift, InterVar, VarSome, GWAVA	4000+
NONCODE (Zhao, Y., et al., 2016)	A dedicated database to non-coding RNAs in 17 species	Non-coding RNA database	VEP, ANNOVAR, SnpEff & SnpSift, VarSome	1000+
TCGA (The Cancer Genome Atlas Research Network, 2019)	A comprehensive atlas that maps key genomic changes in various types of cancer	Cancer genomics	VEP, ANNOVAR, SnpEff & SnpSift, VarSome	10000+
GTEx (Genotype-Tissue Expression) (GTEx Consortium, 2015)	A comprehensive public resource for tissue and cell-specific gene expression and regulation	Gene expression	VEP, ANNOVAR, SnpEff & SnpSift, FUMA, OpenTargets Genetics, OpenCRAVAT	5000+
dbSNP (Sherry et al., 2001)	Largest public archive for genetic variation within and across different species	Variant database- SNP database	VEP, ANNOVAR, SnpEff, SeattleSeq, ShAn, InterVar, VAT, VAAST 2.0, FUMA, HaploReg, OpenCRAVAT, GWAVA, VarSome	7000+
COSMIC (Tate et al., 2019)	The most detailed and comprehensive resource for exploring the effect of somatic mutations in human cancer	Cancer Variant database	VEP, ANNOVAR, SnpEff, InterVar, OpenCRAVAT, VarSome	4000+
ClinVar (Landrum et al., 2014)	An archive with reports and annotations of the relationship between important variants and clinical phenotypes	Clinical Variant database	VEP, ANNOVAR, SnpEff, InterVar, OpenCRAVAT, VarSome	4000+
GWAS Catalog (Buniello et al., 2019)	A comprehensive and highly curated collection of all published GWAS studies	GWAS database	VEP, ANNOVAR, MAGMA, FUMA, OpenTargets Genetics	3,000+
HGMD (Stenson et al., 2003)	A public disease and gene-specific database to obtain both functional and clinical validation of genetic variants	Inherited disease database	VEP, ANNOVAR, SnpEff, InterVar, OpenCRAVAT, VarSome	3000+
ExAC browser (Karczewski et al., 2017) -gnomAD (Karczewski et al., 2020)	A resource developed with the goal to aggregate and harmonize exome and genome sequencing data from a wide variety of large-scale sequencing projects	Population Variant database	VEP, ANNOVAR, SnpEff, OpenCRAVAT, VarSome	2000+
NCI60 (Shoemaker, 2006)	A panel of allele frequency information from 60 cell lines based on their exome sequencing data	Cancer Research database	VEP, OpenCRAVAT	1500+
dbNSFP (Liu et al., 2020)	A repository with all potential non-synonymous single-nucleotide variants (nsSNVs) in the human genome	Variant - Functional Annotation database	VEP, ANNOVAR, SnpEff, OpenCRAVAT, VarSome	1000+
Clingen (Rehm et al., 2015)	A database developed to improve understanding of genomic variation and its use in clinical care	Clinical Variant database	InterVar	1000+
GWAS Atlas (Watanabe et al., 2019)	A database of publicly available GWAS summary statistics	GWAS database	FUMA	500+
FANTOM-FANTOM6 (Ramilowski et al., 2020)	A global project that aims to identify functional elements in mammalian genomes, focusing on non-coding RNAs (especially lncRNAs) in humans. It also provides atlases of mammalian promoters, enhancers, lncRNAs, and miRNAs	Functional Genomics	FUMA, HaploReg	500+
LncRNA2Target v3.0 (Cheng et al., 2019)	A database that contains the most complete lncRNA-Target relationships to date, by reviewing all published lncRNA papers	lncRNA Target database	-	300+
dbscSNV (Jian et al., 2014)	A database of all human SNVs within splicing consensus regions and their functional annotations	Splice Site Variant database	VEP, ANNOVAR, SnpEff	200+
ncVarDB (Biggs et al., 2020)	An open-source, manually curated database of well characterized non-coding human genome variants based on published evidence	Non-coding Variant database	VEP, FUMA, HaploReg	100+
Gene4*Denovo* (Zhao et al., 2020)	A database focused on *de novo* mutations (DNMs) from WES and WGS data and prioritization of candidate genes	*de Novo* Mutation database	VEP, ANNOVAR	100+
lncRNASNPv3 (Yang et al., 2023)	A comprehensive database for functional annotation of variants in long non-coding RNAs	lncRNA Variant database	-	50+

### 4.1 Resources of functional genomics

The Encyclopedia of DNA Elements (ENCODE project) (ENCODE Project Consortium, 2012) is the largest and most comprehensive resource for functional genomics, providing extensive information on regulatory elements across the human genome. ENCODE data encompasses TFBS, histone modifications, chromatin accessibility, and RNA transcripts, which are crucial for understanding gene regulation. Popular variant annotation tools such as VEP, ANNOVAR, SnpEff & SnpSift, FUMA, OpenTargets Genetics, and GWAVA integrate ENCODE data to enhance the functional annotation of genetic variants. This integration allows these tools to predict the impact of variants on gene expression and regulatory mechanisms, offering valuable insights into their potential roles in health and disease. By leveraging ENCODE rich dataset, these tools provide more accurate and context-specific variant annotations, facilitating the identification of disease-associated variants and aiding in the interpretation of complex genomic data.

Other useful resources and projects, with comprehensive functional annotations for variants from both protein coding and non-coding regions are the GENCODE (Harrow et al., 2012), TCGA (Cancer Genome Atlas Research Network et al., 2013), GTEx (GTEx Consortium, 2013) and RefSeq (Pruitt et al., 2014), GWAS Atlas (Watanabe et al., 2019).

GENCODE offers high-quality gene annotations, including protein-coding genes, non-coding RNAs, and pseudogenes. Its extensive gene models are essential for variant annotation tools such as VEP, ANNOVAR, and SnpEff, which utilize GENCODE data to forecast the impact of variants on gene function and structure, especially within non-coding regions and regulatory elements.

TCGA is a cancer genomic project that incorporates genomic datasets on different cancer types, including somatic mutations, gene expression, and epigenetic modifications. It is widely used by tools such as VEP, ANNOVAR, and OpenCRAVAT for functional annotation of cancer-related variants and identification of potential biomarkers and therapeutic targets related to TCGA data.

GTEx offers data on tissue-specific gene expression and regulatory variants, enabling researchers to understand how genetic variation influences gene expression across different tissues. Annotation tools like VEP, ANNOVAR, FUMA, OpenTargets Genetics or HaploReg use GTEx data to link variants with expression quantitative trait loci (eQTLs), aiding in the functional interpretation of non-coding variants and their tissue-specific effects.

RefSeq is a comprehensive database that provides a curated collection of reference sequences for the human genome, including genes, transcripts, and proteins. It serves as a foundational database for annotating variants and understanding gene function. Annotation tools like ANNOVAR, VEP, and SnpEff utilize RefSeq data to predict the functional impact of genetic variants, ensuring accurate and standardized variant interpretation across studies.

GWAS Atlas is a comprehensive collection of GWAS results integrating data from multiple studies. It is widely applied by tools such as VEP, FUMA or MAGMA to prioritize and annotate variants associated with complex traits, facilitating the identification of potential causal variants and genes.

### 4.2 Resources of population and allele frequency data

Population and allele frequency data are critical resources for understanding genetic diversity, identifying rare and common variants, and interpreting the clinical significance of genetic variants. A number of large-scale genomic databases and projects provide comprehensive data on allele frequencies across different populations, playing a crucial role in elucidating the genetic architecture of populations. Genetic architecture refers to the characteristics of genetic variation that are responsible for broad-sense phenotypic heritability, such as the variants influencing a phenotype, the magnitude of their effects, their population frequency and their interactions with each other and the environment (Timpson et al., 2018). The population frequency of variants is important for assessing their potential impact, as rare variants are more likely to have a larger contribution to disease, whereas common variants are expected to have lower effect sizes. Therefore, the allele frequency information helps in identifying population-specific rare variants that may have significant functional impact and thus reducing spurious and non-causal associations by filtering out the multitude of low-impact variants. Nevertheless, genetic architecture information must be used holistically for proper population stratification, as spurious associations may arise from other confounding effects. For instance, if a particular variant is more common in a subpopulation that also has a higher prevalence of a certain phenotype, statistical analyses may incorrectly infer a causal relationship between the variant and the phenotype, especially if the study is underpowered. Consequently, proper adjustment is often needed for population structure, such as using principal component analysis or mixed models (Sul et al., 2018).

The 1000 Genomes Project Project (Genomes et al., 2015), provides an extensive catalog of human genetic variation by sequencing genomes from a diverse array of individuals. This repository includes allele frequency data, which aids in identifying both rare and common variants across different populations. Annotation tools such as VEP, ANNOVAR, and SnpEff utilize this dataset to provide insights into population-specific variant effects, thereby facilitating research into the genetic foundations of complex traits and diseases.

ExAC browser (Karczewski et al., 2017) and its successor gnomAD (Karczewski et al., 2020) provide extensive allele frequency data from diverse populations, facilitating the assessment of pathogenicity of rare variants within populations. Annotation tools like VEP, ANNOVAR, and SnpEff integrate gnomAD data to assess the population frequency of variants, helping researchers distinguish between benign and potentially pathogenic variants.

### 4.3 Clinical and disease-related databases

Clinical and disease-related variant databases are essential resources for understanding the clinical significance of genetic variants and their associations with various diseases.

ClinVar (Landrum et al., 2014) and dbSNP (Sherry et al., 2001) include extensive collections of clinically relevant variants and single nucleotide polymorphisms, respectively, contributing to the understanding of variant pathogenicity and population frequencies. ClinVar is a premier database for variant-clinical significance, providing annotations on the relationship between genetic variants and clinical conditions.

dbSNP (Sherry et al., 2001) is a public archive particularly suited for SNPs and large-scale functional annotation of variants within and across different species. It provides essential data for variant annotation tools such as VEP, ANNOVAR, SnpEff, and FUMA, enabling them to annotate known polymorphisms and assess their potential impact based on population frequency and functional predictions.

Other useful databases aiming to provide clinical or disease variant information: COSMIC (Tate et al., 2019), Human Gene Mutation Database (HGMD) (Stenson et al., 2003), GWAS Catalog (Buniello et al., 2019), NCI60 (Shoemaker, 2006), Clingen (Rehm et al., 2015), dbscSNV (Jian et al., 2014), dbNSFP (Liu et al., 2020), Gene4*Denovo* (Zhao et al., 2020).

COSMIC (Tate et al., 2019) focuses on somatic mutations in cancer, providing detailed mutation data across various cancer types. A variety of functional annotation tools such as VEP, ANNOVAR, SnpEff, InterVar utilize COSMIC data to annotate cancer-related variants.

HGMD (Stenson et al., 2003) is a comprehensive gene mutation database associated with human diseases, providing a valuable resource for clinical variant interpretation. Functional annotation tools such as VEP, ANNOVAR, InterVar use HGMD data to annotate pathogenic variants and discover novel disease target genes.

GWAS Catalog (Buniello et al., 2019) a widely used genome-wide association studies resource, linking genetic variants to complex traits and diseases. It is used by different genome wide annotation tools such as VEP, ANNOVAR, FUMA, MAGMA and Open Targets Genetics to prioritize variants based on their association with traits, facilitating the identification of disease-associated loci and the exploration of their functional consequences.

NCI60 (Shoemaker, 2006) a database for genomic and pharmacological data on 60 different human tumor cell lines aiding in the exploration and study of cancer biology and drug response.

Clingen (Rehm et al., 2015) curates clinically relevant genes and variants providing annotation for clinical variants interpretation. It is widely applied by tools such as VEP, ANNOVAR, SnpEff, InterVar, VarSome to classify variants according to their clinical significance, by translating genomic into clinical data.

dbscSNV (Jian et al., 2014) includes all potential human SNVs within splicing consensus regions, providing functional annotations for splicing variants. It is used by VEP, ANNOVAR, and SnpEff.

dbNSFP (Liu et al., 2020) is a comprehensive database for functional predictions and annotations of non-synonymous and splicing variants. It aggregates data from multiple prediction tools, which are used by VEP, ANNOVAR, and SnpEff to provide detailed functional impact assessments for coding variants, enhancing the accuracy of variant interpretation.

Gene4*Denovo* (Zhao et al., 2020) database focuses on *de novo* mutations and their association with diseases, providing comprehensive variant-level and gene-level annotation and information regarding the DNMs and candidate genes. ANNOVAR uses Gene4*Denovo* data to annotate *de novo* variants, aiding in the discovery of novel disease-causing mutations.

Each database has a different focus and is therefore best suited for specific research or clinical applications. For instance, ClinVar and Clingen are intended for clinically-oriented interpretation of variants. However, ClinVar serves as a central repository for variant data, often including details on phenotype associations, submission evidence, and any conflicting classifications. Because multiple laboratories contribute to ClinVar, the database can contain varying levels of evidence and sometimes conflicting interpretations for the same variant. ClinGen on the other hand is an expert-driven initiative focused on improving the quality and consistency of variant and gene interpretations. dbSNP offers broad coverage of known variants and is often used as a first check to see if a variant has been reported, though it offers limited clinical details. COSMIC specializes in somatic mutations in cancer and is appropriate for oncology research, identifying tumor-specific variants, and understanding cancer mutation spectra. NCI60 is used for pharmacogenomics applications in oncology. dbscSNV and dbNSFP are research-oriented, as they both aggregate *in silico* predictions of functional impact of splicing and non-synonymous variants, respectively.

### 4.4 Non-coding variation resources

NONCODE (Zhao et al., 2016) is a widely cited and comprehensive resource focusing on non-coding variants. It is integrated by popular variant annotation tools such as VEP, ANNOVAR, SnpEff & SnpSift, VarSome to enhance functional annotations for non-coding variants. NONCODE specializes in cataloging non-coding RNAs (ncRNAs), including lncRNAs, microRNAs, and small nuclear RNAs, providing extensive information on their sequences, structures, and functional annotations. The database integrates data from various sources to offer a detailed overview of ncRNA biology, including expression patterns, interactions, and regulatory roles.

Other useful databases and resources for the annotation of variants in non-coding regions are FANTOM-FANTOM6 (FANTOM Consortium and the RIKEN PMI and CLST DGT et al., 2014; Ramilowski et al., 2020), lncRNASNPv3 (Yang et al., 2023), ncVarDB (Biggs et al., 2020), LncRNA2Target v3.0 (Cheng et al., 2019).

FANTOM provides data on gene expression and functional regulatory elements, particularly focusing on enhancer activity. FANTOM6 ((FANTOM Consortium and the RIKEN PMI and CLST DGT et al., 2014; Ramilowski et al., 2020)) aims to systematically clarify the roles of long non-coding RNAs (lncRNAs) in human genome.

lncRNASNPv3 (Yang et al., 2023) is a novel database for comprehensive annotation of variants in long non-coding RNAs (lncRNAs), including their potential functional impacts and their roles in disease.

ncVarDB (Biggs et al., 2020) is a manually curated repository for non-coding variants that provides functional annotations and predictions. Tools such as VEP, FUMA and HaploReg utilize ncVarDB data to interpret the effects of non-coding variants, aiding in the understanding of their roles in gene regulation and disease.

LncRNA2Target v3.0 (Cheng et al., 2019) is a novel database for long non-coding RNAs (lncRNAs) including their potential functional impacts. It contains the most complete lncRNA-Target relationships by manually reviewing all published lncRNA articles. Various tools are using it to annotate lncRNA variants and predict their functional impact.

Each resource serves a distinct purpose in the area of non-coding RNA research. NONCODE is a broad repository for non-coding RNA data, while FANTOM provides large-scale transcriptome and functional annotation data, with particular emphasis on genome-wide promoter and enhancer elements crucial for non-coding RNA expression. On the variant-centric side, ncVarDB specializes in annotating and interpreting the functional impact of non-coding variants, while ncRNASNP focuses on SNPs that affect binding sites and secondary structures of non-coding RNAs. Finally, LncRNA2Target focuses on elucidating the relationships between long non-coding RNAs and their target genes, offering curated insights into lncRNA-mediated regulatory pathways.

## 5 Challenges and future directions

### 5.1 Harmonization of functional annotation diversity at the genome-wide level

Overall, a wide range of tools and resources is available, each with its own methods, strengths, and areas of focus. However, this variety creates a fragmented landscape where different tools may offer differing predictions for the same variant, complicating the integration and interpretation of results. Integrating heterogeneous tools and resources for functional variant annotation presents significant challenges, especially when accounting for non-coding and intergenic regions. Several resources rely their functional annotation methods on protein coding regions and focus on exons which are only the 1% of the genome (Sun et al., 2024).

**FIGURE 3:**
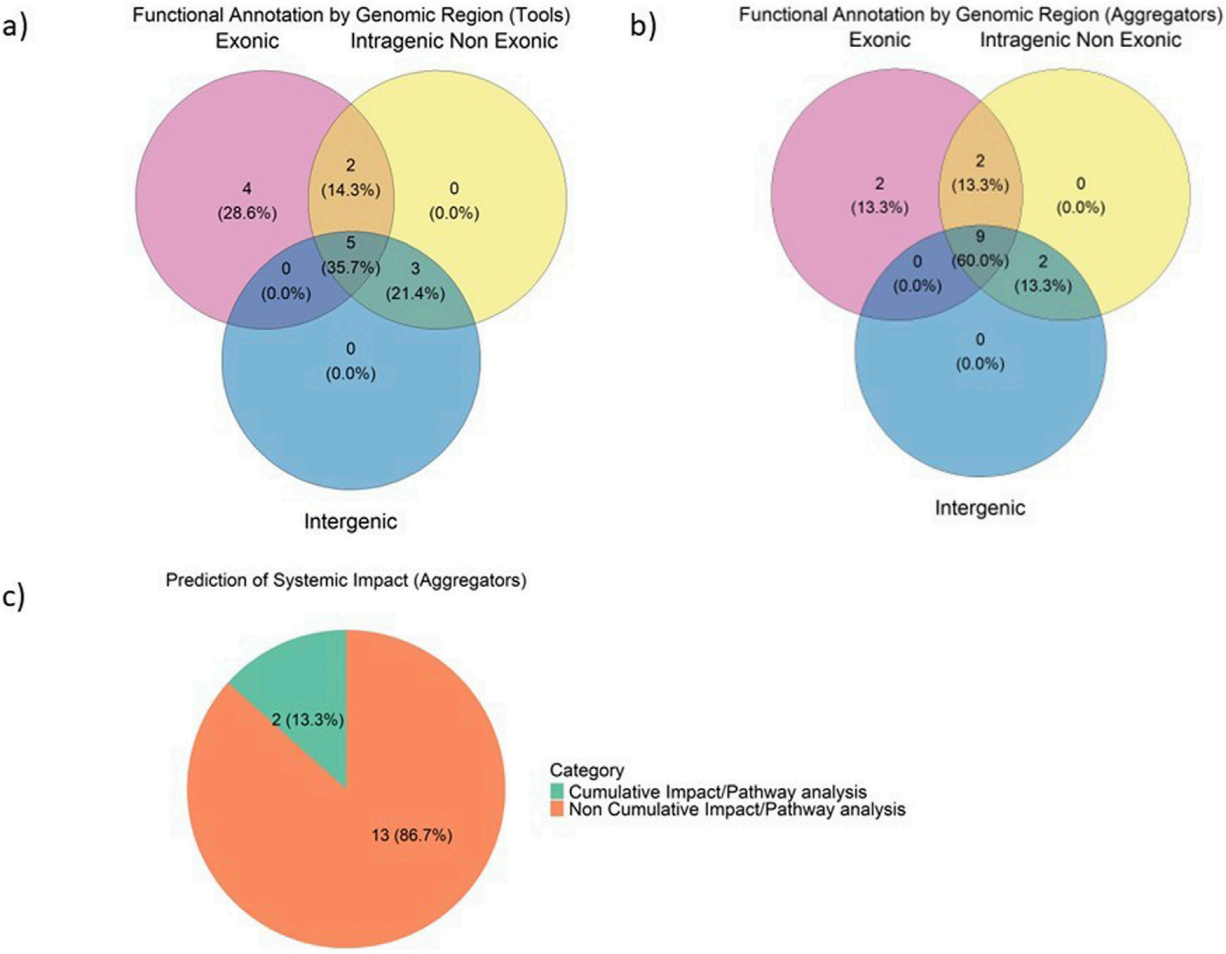
Classification of functional annotation tools **(A)** Venn diagram of annotation tools, classified by genomic region of focus **(B)** Venn diagram of aggregator platforms, classified by genomic region of focus **(C)** Proportions of aggregator tools based on the prediction of the systemic impact of variants.

The Venn diagrams in Figure 3 illustrate the classification of functional annotation tools and aggregators based on their target genomic regions: exonic, intragenic non-exonic, and intergenic. Regarding foundational tools (Figure 3A), 5 tools (35.7%) are classified as genome-wide, as they cover all three genomic regions, reflecting a significant effort towards whole-genome variant interpretation. This trend is even more pronounced among aggregators (Figure 3B), with 9 of them (60%) encompassing the genome in its entirety. Genome-wide coverage is expected to rise, as with the advent of whole-genome studies such as GWAS, it has become evident that non-coding regions play a significant role in many diseases. Deriving biological insight from GWAS is hindered by the fact that the signal mainly lies in intergenic and intronic regions (Giral et al., 2018) and gene causality cannot be inferred with confidence. However, most genetic variation is found in these non-coding areas, highlighting their importance in genomics (Zhang and Lupski, 2015; Giral et al., 2018).

Valuable functional insights for these intergenic regions come from comprehensive functional genomics resources (Table 3). Regulatory elements, such as non-coding RNAs, chromatin regulators and modifiers, enhancers and silencers, play vital roles in the functional annotation of these regions. These elements are integral to the complex regulatory networks that govern gene expression, chromatin structure, and overall genomic function. Grasping these regulatory components is essential for elucidating how genetic variation affects phenotypic diversity and disease susceptibility, making the functional annotation of intergenic regions a pivotal focus in genomics research. Resources such as ENCODE, GENCODE, NONCODE, GTEx, FANTOM, LncRNA2Target, ncVarDB, and lncRNASNPv3 are particularly valuable for this purpose. They provide extensive data and tools for annotating and understanding the functional implications of genetic variants in intergenic and non-coding regions, thereby supporting a deeper comprehension of the genome’s regulatory landscape.

A significant challenge lies in the integration of various functional genomics resources, each of which often focuses on specific aspects of genomic function, such as chromatin accessibility, transcription factor binding, histone modifications, gene expression, and regulatory motifs. Combining these diverse datasets enables a comprehensive view of the genome, capturing a broad range of functional elements and interactions that might be overlooked when relying on a single resource. For instance, ENCODE provides extensive data on transcription factor binding and chromatin states, while GTEx offers valuable information on tissue-specific gene expression and expression quantitative trait loci (eQTLs). FANTOM contributes data on active enhancers and promoters, and NONCODE focuses on non-coding RNAs. Integrating these resources enables researchers to piece together complex regulatory networks and understand how various elements interact to regulate gene function. Genetic variants, especially those in non-coding regions, can have subtle but significant effects on gene regulation. Integrating multiple functional genomics datasets enhances the ability to interpret these variants’ functional impacts. For example, a variant might disrupt a TFBS identified by ENCODE, alter a histone modification state from Roadmap Epigenomics data, or affect gene expression as shown by GTEx eQTL data. By considering all these aspects, researchers could better predict the potential phenotypic consequences of genetic variants.

Consequently, the integration of diverse functional genomics resources requires novel advanced bioinformatic methodologies, such as network-based approaches and systems biology frameworks. These sophisticated analyses require rich, multi-dimensional and harmonized data to generate accurate and meaningful predictions. It also entails developing interoperable tools capable of seamlessly integrating multiple data sources, ensuring that the functional impacts of variants across all genomic regions are comprehensively captured and interpreted. This level of integration is vital for enhancing our understanding of the genome and propelling research into complex genetic traits and diseases.

### 5.2 Systemic and cumulative impact of genetic variants

Understanding the cumulative impact of genetic variants and polygenic associations presents a significant challenge in genomics (Lewis and Vassos, 2020). Most variants identified through GWAS individually have small effect sizes, making it difficult to discern their impact on phenotypes. Unlike rare variants with large effects that can be more straightforwardly linked to monogenic traits or diseases, common variants require large sample sizes to detect significant associations with complex diseases. Moreover, the effects of individual variants may be influenced by interactions with other genetic variants (epistasis) and with environmental factors. These interactions can either amplify or mitigate the effects of individual variants, adding another layer of complexity to understanding their cumulative impact. However, evidence of epistasis at the level of variants remains limited (Balvert et al., 2024).

Furthermore, molecular pathways and complex interactions at the gene or protein level are critical components of cellular and organismal function, and they offer a compelling framework for understanding how genetic variants can cumulatively impact phenotypes. The integration of genetic variation with molecular interaction networks and biological pathways helps elucidate the mechanisms through which multiple variants collectively influence complex traits and diseases. Functional analysis at the pathway level, leveraging functional gene annotation (Maleki et al., 2020), is a promising approach to understand the biological significance of genetic variants by examining their collective impact on predefined sets of genes, directly (protein-coding regions) or indirectly (colocalization with intergenic regulatory regions).

As shown in Table 1 and Figure 3C, currently, a number of tools are available that integrate gene set enrichment and pathway analysis with genome-wide variant annotation, including MAGMA (de Leeuw et al., 2015) and FUMA (Watanabe et al., 2017). MAGMA is a stand-alone tool that performs gene-set analysis by first aggregating SNP-level associations into gene-level statistics and then assessing the enrichment of association signals within predefined gene sets. These gene sets can be collected by the MSigDB database (Subramanian et al., 2005) or defined by the user using any gene-to-pathway mappings, e.g., from (Kanehisa and Goto, 2000), Reactome (Milacic et al., 2024) or Gene Ontology (GO) (Ashburner et al., 2000; The Gene Ontology Consortium, 2019), thus depending on static, literature-curated collections of biological pathways as a major input. FUMA is a web-based platform that incorporates MAGMA to perform gene-based tests and aggregating SNP-level associations to identify significant genes, integrating multiple biological repositories and tools to process GWAS summary data, offering a comprehensive platform for post-GWAS analysis. Therefore, FUMA also incorporates variants located at intergenic regions, by leveraging functional genomic resources. A limitation to the MAGMA method is that it relies on gene sets, without performing semantic analysis in order to leverage the topological properties of semantic graphs such as the biomedical ontologies.

The category of tools specifically designed to assess the cumulative impact of multiple variants on pathways or biological processes was found under-represented under our review criteria. Nevertheless, as stated before, this review focuses on most recently published and highly cited tools, representing most common practices and current trends. Examples of additional pathway-based approaches include INRICH (Lee et al., 2012; Wang et al., 2007), both leveraging GSEA (Subramanian et al., 2005). Methods capable of evaluating variant collective effects are essential to fully understand the genetic architecture of polygenic diseases. Pathway-level functional interpretation is essential not only for the harmonization of the annotations, but also for their prioritization, especially since each genome contains thousands of variants that differently influence phenotypes. Enhancing and expanding this category of tools is essential for advancing our comprehension of how combinations of variants contribute to complex phenotypes and for facilitating the development of targeted therapeutic strategies.

### 5.3 Towards genome-wide, standardized, systems-level interpretation and prioritization

It is essential to develop novel approaches that enhance confidence in identifying causal genes affected by genomic variation and elucidate the pathways underlying genetic association signals. These approaches should be capable of leveraging genome-wide functional annotations for intronic and intergenic variants and pinpointing causal genes that directly impact non-coding regulatory elements. (Uffelmann et al., 2021). Until now enhancers and promoters are the most studied regulatory elements but there is plenty of space in other elements such as non-coding RNAs or transposable elements (Hadjiargyrou and Delihas, 2013).

A primary challenge lies in developing robust pipelines, workflows, and tools that ensure reproducible results and remain robust despite minor changes in parameterization. Existing tools, while leveraging a vast array of databases for annotation, often retrieve substantial amounts of false or noisy information, which limits both the accuracy and effectiveness of the annotation process. Therefore, reproducibility and standardization are of paramount importance to minimize unavoidable noise and extract meaningful information. An additional limitation regarding scalability includes the management and storage of the resulting data (Zhou et al., 2023).

On this scope, semantic and network-based processing is a promising direction towards standardized interpretation of whole-genome and genome-wide association studies. Semantic processing using biomedical ontologies, such as the Gene Ontology (GO), offers significant advantages for the standardization of the interpretation, enhancing the reliability, reproducibility, and depth of insights.

Semantic processing frameworks may facilitate the systemic understanding of the overall impact that vectors of genomic variants have on their hosts. This includes understanding how these variants relate to the risk of manifesting distinct phenotypes or pathologies. Digital solutions that can analyze these variant vectors holistically, leveraging various semantic sources such as ontological vocabularies, gene set mappings, and interaction graphs, could provide a rational basis for data-agnostic, systems-level comparative evaluations across different hosts. Such approaches would pave the way for precise, data-driven diagnostic and therapeutic stratification. They may also facilitate the selection of animal models, such as mice and rats, that functionally align with specific phenotypic aspects of human diseases by enabling comparisons at the functional and pathway levels, as individual gene expression responses have been shown to poorly translate between the two species (Seok et al., 2013).

## 6 Conclusion

The development of analytical pipelines that standardize multi-layered genome-wide functional annotation and biological interpretation, including the intergenic regions of the human genome, may enable the optimal utilization of the rapidly growing volume of high-throughput genomic data.

Functional annotation of intergenic regions is crucial for understanding the regulatory roles and impact of genetic variants outside protein-coding regions. For instance, complex traits, in contrast to Mendelian diseases, are driven by non-coding variants that affect gene regulation (Welter et al., 2014; Li et al., 2016). Various functional genomics resources provide crucial data for annotating these regions, aiding in comprehending gene regulation and phenotypic diversity. Integrating these diverse resources offers a comprehensive view of the genome, which is vital for systematically interpreting the cumulative, synergistic or antagonistic, effects of non-coding variants on complex diseases and phenotypes. However, a standardized and universal framework for integrating, interpreting and prioritizing all variants across the genome is still lacking.

Whole-Genome and GWAS studies, which provide significant amounts of data and genetic associations to diseases, have attracted criticism for their limited utility when it comes to their meaningful interpretation into real-life clinical applications (Tam et al., 2019; Boyle et al., 2017). However, GWAS remains an effective tool and a valuable source of data the utility of which is further enhanced with the exploitation of various techniques and approaches, such as fine mapping and pathway or gene-set level interpretation, aiming at narrowing down the set of candidate variants.

In addition, advanced pathway-level analysis provides a means to address annotation discrepancies, another common issue in variant interpretation. Divergence, variation and even conflicting data have been observed in virtually all aspects of genomic analysis: sequencing technologies and technical variation, variant calling algorithms, annotation tools and algorithms for variant effect prediction (e.g., ANNOVAR, VEP), reference genome assemblies (GRCh37 vs. GRCh38), gene annotation resources (e.g., RefSeq, Ensembl) and variant databases (e.g., ClinVar, ClinGen, dbSNP). Standardized, systems-level approaches that integrate functional and regulatory information with semantic-based biological interpretation may offer a rigorous foundation for data-driven comparative analysis of genetic fingerprints and further support precise diagnostic, epidemiological, and therapeutic strategies.
